# Depression in Primary Care Patients with Coronary Heart Disease: Baseline Findings from the UPBEAT UK Study

**DOI:** 10.1371/journal.pone.0098342

**Published:** 2014-06-12

**Authors:** Paul Walters, Elizabeth A. Barley, Anthony Mann, Rachel Phillips, André Tylee

**Affiliations:** 1 University Department of Mental Health, Dorset HealthCare University NHS Foundation Trust and Bournemouth University, Bournemouth, Dorset, United Kingdom; 2 Florence Nightingale School of Nursing and Midwifery, King's College London, London, United Kingdom; 3 Section of Primary Care Mental Health, Health Services and Population Research Department, King's College London, London, United Kingdom; S.G.Battista Hospital, Italy

## Abstract

**Background:**

An association between depression and coronary heart disease is now accepted but there has been little primary care research on this topic. The UPBEAT-UK studies are centred on a cohort of primary patients with coronary heart disease assessed every six months for up to four years. The aim of this research was to determine the prevalence and associations of depression in this cohort at baseline.

**Method:**

Participants with coronary heart disease were recruited from general practice registers and assessed for cardiac symptoms, depression, quality of life and social problems.

**Results:**

803 people participated. 42% had a documented history of myocardial infarction, 54% a diagnosis of ischaemic heart disease or angina. 44% still experienced chest pain. 7% had an ICD-10 defined depressive disorder. Factors independently associated with this diagnosis were problems living alone (OR 5.49, 95% CI 2.11–13.30), problems carrying out usual activities (OR 3.71, 95% CI 1.93–7.14), experiencing chest pain (OR 3.27, 95% CI 1.58–6.76), other pains or discomfort (OR 3.39, 95% CI 1.42–8.10), younger age (OR 0.95 per year 95% CI 0.92–0.98).

**Conclusion:**

Problems living alone, chest pain and disability are important predictors of depression in this population.

## Introduction

In the United Kingdom, General Practitioners (GPs) receive payments for chronic disease management of patients with coronary heart disease (CHD) and for screening these patients for depression. This is because a possibly bi-directional association between depression and CHD is now accepted [1], [2]. CHD registers are thus held in general practice [3], but little is known about the characteristics of those placed on these registers. Despite this required primary care activity, the published research that suggests the link between CHD and co-morbid depression has been conducted mainly on patients post cardiac event, recruited in secondary care. Patients with CHD have been reported to be at an increased risk of suffering from depression compared to age matched controls [4]–[6]. It has also been reported that depression increases all cause mortality in patients with CHD [7], and that developing depression following an acute myocardial infarction increases cardiac mortality [8]. Pajak *et al* explored the prevalence of depression in patients following hospitalisation for coronary heart disease across Europe and found a prevalence of between 8.2% and 35.7% in men and 10.3% to 62.5% in women, depending on country, with a prevalence in the United Kingdom of 19.4% in men and 17.5% in women. [9]

While the relationship between CHD and depression may be bi-directional as suggested by these studies, it is not known whether any relationship is maintained as the cardiac event becomes distant in time. Is there a persisting increased risk of depression, for example, in those with a known history of CHD, regardless of current symptoms or disability? Do those with recurrent or persistent depression have more disabling cardiac morbidity or a greater risk of a further cardiac event? If the relationship persists, then an underlying biological mechanism linking them becomes more likely – shared genetic risk and/or enhanced inflammatory response are currently being researched [10].

More could be elucidated with longer-term follow up of less selected populations. Depression, anxiety and coronary heart disease are common amongst consulting patients. The prevalence rate of depression was 10.4% in consecutive attenders across centres participating in the World Health Organisation's Psychological Problems in General Health Care study [11]. Coronary heart disease is also common in primary care attenders with a prevalence rate of 8% in men and 5% in women over the age of 44 years [12].

The primary care CHD register is an available resource that could be used to explore these questions. The UPBEAT-UK research programme was set up in 2007 and consists of qualitative and quantitative studies to determine the prevalence of depression and anxiety in primary care patients with CHD, to explore the relationship between these diagnoses and continued cardiac symptoms, new cardiac morbidity and mortality [13]–[15]. At its core is a cohort study of 803 patients recruited from primary care CHD registers in 16 practices in South London. Participants are followed up every six months for up to four years so that relationships between changes in physical and mental health can be tracked thus furthering our knowledge of the direction of causality. Also as part of this programme of research a pilot randomised controlled trial to improve depression outcomes for primary care patients with depression and CHD is also underway [16].

The aims of this research were to describe the socio-demographic and clinical characteristics of the recruited population with CHD and determine the prevalence rate of depression and factors associated with depression in this population.

## Methods

Details of the cohort study protocol have been reported elsewhere [13]. The sampling frame comprised all people on the Quality and Outcomes Framework Coronary Heart Disease (CHD) Registers kept by participating general practices [3]. The Greater London Primary Care Research Network recruited sixteen General Practices from inner city and suburban south London. All patients on the participating GPs' CHD registers were sent an ‘invitation to participate’ letter by their GP. Recruitment and baseline assessments were completed during 2008-9.

### Ethics Statement

Written, informed consent was obtained for all participants before the initial assessment was conducted. Ethical approval was granted through the Bexley and Greenwich Research Ethics Committee (REC reference number: 07/H0809/38).

### Measures

Details of measures used have been reported in full [13]. The Rose Angina Questionnaire [17] was used to assess the presence and symptoms of chest pain at inclusion into the cohort. Depression and anxiety were assessed using the Clinical Interview Schedule-Revised (CIS-R) [18]. This yields International Classification of Diseases-10 (ICD-10) [19] diagnoses for depression and anxiety and also assesses the severity of these conditions. In addition, participants completed the Hospital Anxiety and Depression scale (HADS) [20], those scoring 8 or more being identified as probable cases of depression. Quality of life was measured using the EQ-5D [21] and current social problems using the Social Problem Questionnaire (SPQ) [22]. GP records of participants were anonymised and then reviewed by clinical members of the research team to collect information on coronary heart disease status and current and past medical diagnoses including depression and anxiety. The prevalence rate of current coded diagnoses of depression in the notes represents, in the Goldberg-Huxley model, the conspicuous psychiatric morbidity in this population [23]. Participants were either assessed at home or at GP surgeries according to their preference.

### Statistical analyses

Data were analysed using Stata 11.2 (StataCorp, Texas). Means and standard deviations were used to summarise normally distributed continuous data. Non-normally distributed continuous data were summarised using medians and range. Categorical data were summarised using both the number and proportion.

The primary outcome was meeting criteria for a CIS-R diagnosis of a depressive disorder or having no such diagnosis. Logistic regression was used to calculate unadjusted odds ratios (ORs) for associations between predictor variables and outcome and then to develop parsimonious multivariate models of predictors for depression both as identified by CIS-R and through diagnostic codes in the medical notes as a current problem. Two-sided 5% significance level was used for all analyses.

## Results

Sixteen practices in South East and South West London participated in the study. The total practice population was 142,648 patients; of this population 2% (2938/142,648) were listed on the QOF CHD registers. Thirty one per cent (n = 917) of the latter, after invitation by a letter from their GP to participate in the study, agreed to be contacted by the research team; 88% (803/917) were then interviewed and enlisted into the cohort for follow up. The study population therefore represents 27% (803/2938) of those on the CHD registers.

The mean age of participants was 71 years (standard deviation (s.d.)10.9). Seventy per cent were male and 87% were white. The mean Index of Multiple Deprivation Score for the cohort was 20.3 (s.d. 14.0). The psychiatric status was as follows: 19% (149/803) met the criteria for an ICD-10 defined diagnosis of a depressive or an anxiety disorder; 7% (54/803) met criteria for depressive disorder of which 31% (17) were classed as severe; 7% (56/803) were also recorded in the medical notes as having depression as an active, current problem and 3% (26/803) similarly with anxiety or anxiety with depression as an active, current disorder. The rate of conspicuous morbidity was thus 10%. Thirteen percent (103/799) scored 8 or more on the HADS depression subscale, thus being classed probable cases of depression by that scale. Multiple social problems and disabilities were reported by participants. Most common were problems with pain and discomfort (53%, 425/803)), mobility (49%, 391/803)) and difficulties with intimate relationships (38%, 302/803).

The cardiac status of participants was as follows: a history of myocardial infarction was documented for 42% (339/803) and 54% (431/803) had a diagnosis of ischaemic heart disease and/or angina; 4% had cardiac diagnoses other than coronary heart disease (or no diagnosis recorded n = 2)). The mean length of time since CHD was first recorded in GPs' notes was 10.4 years (s.d.8, range 6 months to 43 years); 52% (418/803) had undergone a surgical intervention (stent, angioplasty, bypass graft, pacemaker or ablation). Forty four per cent (356/803) reported that they continued to experience chest pain. The frequencies of demographic factors, cardiac, other physical health and social variables and their association with CIS-R depression diagnoses are shown in Tables 1, 2, 3 and 4.

**Table 1:** Socio-demographic characteristics and unadjusted odds ratios for a depressive disorder (as defined by the CIS-R) (N = 803 unless otherwise stated).

Variable	N(%)	Odds Ratio	*p*-value	95% confidence interval
Age in years	70.6 (10.9)*	0.96 (per year)	<0.001	0.93–0.98
Female	242 (30.1)	1.65	0.079	0.93–2.91
*Ethnicity*:				
White	701 (87.3)	1		
Black	33 (4.1)	2.28	0.140	0.76–6.80
Asian	47 (5.8)	3.39	0.004	1.48–7.73
Other	22 (2.7)	1.65	0.508	0.37–7.32
*Employment status (N = 797)*:				
Employed	148 (18.6)	1		
Retired	619 (77.7)	0.68	0.266	0.34–1.34
Unemployed	30 (3.8)	2.27	0.155	0.73–7.00
*Relationship Status (N = 800)*:				
Married/cohabiting	508 (63.5)	1		
Widowed	150 (18.8)	1.04	0.917	0.46–2.36
Separated/divorced	65 (8.1)	4.20	<0.001	2.00–8.80
Single	77 (9.6)	1.57	0.340	0.62–3.94
*Usually live with (N = 800)*:				
Husband/wife/partner	488 (61.0)	1		
Children	33(4.12)	1.30	0.727	0.29–5.79
Alone	236 (29.5)	2.08	0.018	1.13–3.81
Other	43 (5.4)	2.66	0.061	0.95–7.39
*Usual residence (N = 774)*:				
Owner occupier	526 (67.9)	1		
Private rental	53 (6.9)	1.26	0.718	0.36–4.31
Housing association	174 (22.5)	3.03	<0.001	1.65–5.55
Sheltered housing	21 (2.7)	3.49	0.058	0.96–12.65
Index of Multiple Deprivation Score	20.3 (13.9)*	1.02	0.038	1.00–1.04

*Mean (standard deviation).

**Table 2:** Physical Health Status at baseline and unadjusted odds ratio for a depressive disorder (as defined by the CIS-R) (N = 803 unless otherwise stated).

Variable	N (%)	Odds Ratio	*p*-value	95% confidence interval
Reports current chest pain	356 (44.3)	5.44	<0.001	2.76–10.72
*Primary GP Diagnosis*:				
Documented myocardial infarction	339 (42.2)	1		
Ischaemic Heart Disease	374 (46.6)	1.07	0.820	0.60–1.90
Angina	57 (7.1)	0.50	0.356	0.11–2.18
Other (arrhythmias, heart failure, or not specified)	33 (4.1)	0.89	0.874	0.19–3.94
Time since Coronary heart disease diagnosis (years) (N = 782)	10.4 (7.9)*	1.01 (per year)	0.542	0.977–1.05
*Co-morbid medical illnesses*:				
Diabetes Mellitus	200 (24.9)	1.86	0.035	1.04–3.31
Osteoarthritis	134 (6.7)	1.47	0.261	0.75–2.87
Chronic obstructive pulmonary disease	91 (11.3)	2.14	0.034	1.07–4.32
Chronic renal disease	152 (18.9)	0.97	0.936	0.48–1.98
Asthma	65 (8.1)	2.11	0.066	0.95–4.69
Hypertension	445 (55.4)	1.29	0.384	0.73–2.26
Active cancer	96 (12.0)	1.52	0.272	0.72–3.22
*Total number of co-morbid illnesses*:				
0	157 (19.6)	1		
1	265 (33.0)	3.96	0.029	1.15–13.62
2	228 (28.4)	3.87	0.034	1.11–13.53
>2	153 (19.1)	6.00	0.005	1.71–21.02

*Mean (standard deviation).

**Table 3:** Lifestyle Status at baseline and unadjusted odds ratio for a depressive disorder (as defined by the CIS-R) (N = 803 unless otherwise stated).

Variable	N(%)	Odds Ratio	*p*-value	95% confidence interval
*Body Mass Index Classification (N = 781)*:				
Underweight	7 (0.9)	1		
Normal	187 (23.9)	0.24	0.217	0.03–2.30
Overweight	343 (43.9)	0.27	0.245	0.03–2.43
Obese	251 (32.1)	0.75	0.797	0.09–6.49
*Smoking Status*:				
Never	240 (29.9)	1		
Ex-smoker	460 (57.3)	1.05	0.893	0.540–2.027
Current smoker	103 (12.8)	2.13	0.067	0.95–4.78
*Alcohol use (units per week) (N = 801)*:				
0	225 (28.1)	1		
1–10	385 (48.1)	0.53	0.038	0.29–0.97
11–20	105 (13.11)	0.16	0.015	0.04–0.70
>20	86 (10.7)	0.52	0.195	0.19–1.40

**Table 4:** Social problems and disability at baseline and unadjusted odds ratio for a depressive disorder (as defined by the CIS-R) (N = 803 unless otherwise stated).

Variable	N(%)	Odds Ratio	*p*-value	95% confidence interval
Housing problems	43 (5.4)	3.55	0.003	1.56–8.09
Employment problems	73 (0.1)	3.64	<0.001	1.85–7.17
Financial problems	70 (8.72)	3.00	0.003	1.47–6.11
Lack of social contacts	106 (13.2)	4.13	<0.001	2.26–7.54
Problems with relatives	89 (11.1)	2.81	0.002	1.44–5.47
Relationship problems	302 (37.6)	2.38	0.002	1.36–4.16
Problems living alone	29 (3.6)	8.73	<0.001	3.83–19.90
*Disabilities (N = 802)*:				
Mobility problems	391 (48.8)	3.23	<0.001	1.72–6.04
Self-care problems	101 (12.6)	2.66	0.003	1.39–5.09
Problems with usual activities	237 (29.6)	5.96	<0.001	3.28–10.83
Problems with pain or discomfort	425 (52.9)	5.96	<0.001	3.28–10.83
*Number of disability areas(N = 753)*:				
0	289(38.4)	1		
1	180 (23.9)	4.64	0.010	1.45–14.79
>1	284 (37.7)	7.48	<0.001	2.58–21.68

Historical cardiac variables were not associated with current diagnoses of depression, but there was a strong association with currently reporting chest pain. Depression can be seen to be more common in women and in ethnic minority participants and the prevalence reduced with age. Significant associations were: being divorced or separated, living alone, being unable to carry out usual daily activities and being in pain or discomfort. Being disabled in more than 1 domains of the EQ-5D showed an OR of 7.5 for depression. Reported problems in all domains of the SPQ were also strongly associated with depression.

The agreement between coded diagnosis of depression in the medical notes and CIS-R classification was low: of 110 people identified by either means, only 12 were in common. Despite this, unadjusted associations were similar: younger age (OR per year increase in age 0.97, 95% CI 0.95–0.99, *p* = 0.008), divorced or separated (OR compared to married or cohabiting 3.11, 95% CI 1.44–7.76. *p* = 0.004), female sex (OR 1.97, 95% CI 1.14–3.41, *p* = 0.016), unemployed (OR versus paid employment 3.45, 95% CI 1.15–10.38, *p* = 0.027), living alone (OR versus living with spouse 1.98, 95% CI 1.07–3.65, *p* = 0.028), experiencing chest pain (OR 2.21, 95% CI 1.26–3.87, *p* = 0.005), having a current active diagnosis of diabetes (OR 1.9, 95% CI 1.08–3.35, *p* = 0.026) experiencing other pain and discomfort (OR 2.51, 95% CI 1.36–4.62, *p* = 0.003), having housing problems (OR 3.39, 95% CI 1.49–7.71, p = 0.004), financial problems (2.49, 95% CI 1.20–5.18, *p* = 0.015), a lack of social contacts (OR 2.13, 95% CI 1.10–4.10, *p* = 0.025), problems with intimate relationships (OR 2.55, 95% CI 1.47–4.43, *p* = 0.001), problems living alone (OR 2.95, 95% CI = 1.08–8.06), p = 0.035) and being disabled (OR for problems completing usual activities 2.10, 95% CI 1.21–3.66, *p* = 0.009).

The results of the multivariate logistic regressions are shown in Table 5. For a CIS-R diagnosis of a depressive disorder, reporting problems with living alone, being in pain or discomfort, reporting still experiencing chest pain and having difficulty in carrying out usual daily tasks were independently associated with a diagnosis of depression. Increasing age was associated with a decreased odds ratio. For a GP-coded diagnosis of depression, the variables that remained independently associated with depression were being female, younger age, having pain and discomfort, reporting problems in close relationships and having a diagnosis of diabetes mellitus.

**Table 5:** Parsimonious multivariate logistic regression models for associations between predictor variables and CIS-R and GP diagnosed depression (N = 802).

Variable	Odds Ratio	*z-Score*	*p*-value	95% confidence interval
*CIS-R diagnosis of depression*				
Problems living alone	5.49	3.49	<0.001	2.11–13.30
Experiences chest pain	3.27	3.20	0.001	1.58–6.76
Disabled by pain and discomfort	3.39	2.74	0.006	1.42–8.10
Problems carrying out usual activities	3.71	3.94	<0.001	1.93–7.14
Age at entry into the study (per year)	0.95	−3.67	<0.001	0.92–0.98
*GP case note diagnosis of depression*				
Problems with close relationships	2.51	3.08	0.002	1.40–4.52
Diabetes Mellitus	2.01	2.32	0.020	1.11–3.63
Disabled by pain and discomfort	1.95	2.08	0.037	1.04–3.68
Female sex	1.88	2.11	0.035	1.04–3.37
Age at entry into the study (per year)	0.97	−2.98	0.003	0.94–0.98

## Discussion

To our knowledge, this is the first study to measure the prevalence of depression in a primary care population with CHD. The CHD register was shown to be an efficient means to access a community population with documented CHD; only 4% did not have this pathology but had other cardiac conditions. The majority had been diagnosed with CHD for many years and thus provided a picture of older patients (average age 71years) living at home with CHD. However our cohort consisted of only 27% of those on the registers and this should be born in mind when interpreting our result. This reflects the complex opt-in approach, mediated by the GPs, that is required for current primary care research in the UK today. We achieved a similar inclusion rate to another recently published large scale UK primary care study using the same approach [24]. Our cohort was also predominantly male (29.9%). Whilst this may represent a selection bias it also reflects the higher prevalence of amongst men.

We found the combined prevalence rate of depression and anxiety disorders was 19%; 7% met the criteria for depressive disorder as measured by the CISR-R. The prevalence of depression was higher when measured by the HADS with 13% of the population scoring as probable cases of depression. The risk predictors we found for depression are similar to those reported in the general population in other studies. Salokangas & Poutanen reported that risk factors for depression in the general population were physical health problems, physical disability, and poor social support [25]. Brown & Harris previously reported the association between social problems and the onset of depression [26]. These associations were recognized by GPs, practice nurses and patients participating in qualitative studies as part of the UPBEAT-UK programme [15]. However a novel finding, reflecting the nature of this population was that reporting still experiencing chest pain was one of the strongest associations with depression (as measured by the CIS-R) independent of associations with other pains and discomfort. The chest pain could be due to the underlying ischaemic heart disease or be a somatic symptom associated with the concurrent depression or perhaps both. Further analyses of our data will elucidate this.

The prevalence of depressive disorder was lower than previously reported in one US study of people with CHD living in the community. Egede found a prevalence rate of depression in people with CHD of 15% [5]. Possible explanations for the lower prevalence of depression in our study is response bias - patients with co-morbid depression or anxiety may be less likely to respond to the GP's letter inviting participation in the study leading to an underestimation of the prevalence rate, but is also likely to represent the sensitivity of instruments used to detect depression. In our study we used the CIS-R as a ‘gold-standard’ as this generates ICD-10 diagnoses rather than the probability of depression based on symptom scores. When the HADS was used the prevalence of depression was similar to that found by Egede. The effect of response bias cannot be assessed as information on patients not agreeing to participate was not available to us. In the EUROASPIRE study, a prevalence of depression (as measured by the HADS) of 18.5% was found in a population of patients recruited in hospitals in the UK at least 6 months after an index cardiac event (median time a after index event >1 year) [9]. However, this study represents a secondary care population and the number recruited into the study from the UK was relatively small (n = 80) so comparisons with the primary care population in our study are not very applicable.

Comparisons can thus only be tentative. Our total prevalence of depression and anxiety (19%) was in keeping with that reported in the general UK population (18%), but the prevalence rate of depression alone in that study, which also used the CIS-R, was only 2.6% [27]. Given that virtually everyone in the UK is registered with a general practitioner, results of this community survey should be very similar to rates among patients on practice lists. Singleton et al reported the lowest prevalence rate of depression and anxiety disorders was in those aged between 65and 74 years (∼10%) and lowest in men of that age group (5.7%) [27]. The population in our study was predominantly male with a mean age of 71 years, suggesting that our prevalence rate was much higher than that which might be expected in the general community. Another comparator would be prevalence rates among patients listed on GP registers for other physical conditions - diabetes, asthma or hypertension for example. These data appear rare. In one study,114 patients from asthma registers of four practices in Salford, UK were assessed [28]. Depression, defined by scores on the HADS, was present in 10% of the sample, similar to our HADS rate of 12.9% [28]. However other studies have failed to find an increase in the prevalence of depression in people with coronary heart disease [29]. Gulliksson *et al* compared patients with CHD discharged within 1 year of an acute coronary event and found no difference in the prevalence in this population and a matched reference population [29]. Again, care should be taken if making comparisons with this study as they represent very different populations.

The purpose of CHD registers is to allow GPs and practice nurses to check on the health of those listed on them and screening for depression has been required as part of the QOF. This study suggests that depression is probably more frequent in this CHD population than in the general population and importantly this is a finding arising from primary care rather than secondary care research. The positive associations we report can be useful as additional markers of the presence of depression, suggesting those who need particular attention at their routine follow up by practice staff. The discrepancy between patients with a CIS-R diagnosis of a depressive disorder and a GP case record diagnosis of depression could be explained in part by the fluctuating nature of depressive symptoms and that patients weren't assessed using the CIS-R at the same time they received a case record diagnosis; cases recorded in the GP notes may have recovered by the time they were assessed using the CISR-R or indeed deteriorated. Female patients were identified preferentially by GPs, judging by medical notes and reflecting previous studies of GP detection [30]. As the register population is in the majority male, losing that bias and focusing on patients of either sex with the complaints of current experiences of chest pain, being unhappy living alone and having difficulties in coping with daily living would enhance detection of current depression. The relationship we found between diabetes and depression in GP coded depression is likely to reflect the fact that GPs are also remunerated as part of the QOF for screening for depression in patients with diabetes too.

We can say nothing about directions of causality for the associations we report because these are cross sectional data. Nor can our data be generalized in view of the low response rate. The multi-wave follow-up of these study participants will allow associations to be tested in a more substantial way.
